# Coexistence of K-ras mutations and HPV infection in colon cancer

**DOI:** 10.1186/1471-2407-6-115

**Published:** 2006-05-04

**Authors:** Nur Buyru, Ayda Tezol, Nejat Dalay

**Affiliations:** 1 Department of Medical Biology, Cerrahpasa Medical Faculty, Istanbul University, Istanbul, Turkey; 2 Oncology Institute, Istanbul University, Istanbul, Turkey

## Abstract

**Background:**

Activation of the ras genes or association with human papillomavirus infection have been extensively studied in colorectal cancer. However, the correlation between K-ras mutations and HPV in colorectal cancer has not been investigated yet. In this study we aimed to investigate the presence of K-ras mutations and their correlation with HPV infection in colon cancer.

**Methods:**

K-ras mutations were analyzed by a mutagenic PCR assay and digestion with specific restriction enzymes to distinguish the wild-type and mutant codons. HPV infection was analyzed by PCR amplification and hybridization with specific probes by Southern blotting. Stattistical analyses were performed by the chi-square and Fisher's exact tests

**Results:**

HPV gene fragments were detected in 43 tumors and 17 normal tissue samples. HPV 18 was the prevalent type in the tumor tissue. A mutation at codon 12 of the K-ras gene was present in 31 patients. 56% of the HPV-positive tumors also harbored a K-ras mutation. Codon 13 mutations were not observed. These data indicate that infection with high risk HPV types and mutational activation of the K-ras gene are frequent events in colorectal carcinogenesis.

**Conclusion:**

Our findings suggest that mutational activation of the K-ras gene is a common event in colon carcinogenesis and that HPV infection may represent an important factor in the development of the premalignant lesions leading to the neoplastic phenotype.

## Background

Colorectal carcinogenesis is a complex, multistep process involving environmental and lifestyle factors, sequential genetic changes and possibly viral components in discrete geographical areas. Genetic changes inactivating tumor suppressor and mismatch repair genes or activation of oncogenes which are involved in cell growth, proliferation and differentiation are implicated in the development of colon carcinoma. The target genes for these alterations are APC (Adenomatous Polyposis Coli), the ras family and p53 [1]. The ras family of oncogenes (N-ras, H-ras and K-ras) encode a small 21-kD protein (p21 ras) involved in the transduction of external stimuli to effector molecules across plasma membranes [2]. This protein has an intrinsic GTPase activity allowing inactivation following signal transduction in the normal cellular environment [2,3]. Activation of the K-ras protooncogene by point mutation is one of the most frequent genetic alterations associated with human cancers [4,5]. Mutated ras p21 has a structure that disfavors its ability to bind the GTPase activating protein (GAP), thus keeping the p21 in the GTP-bound, activated state [6]. Approximately, 90 % of these activating mutations occur in codons 12 and 13 of exon 1 identifying these codons as hot-spot targets [7]. The incidence of ras gene mutations, however, varies considerably among different types of cancer and the profile of ras oncogene activation is often specific for each tumor type. For example, K-ras mutations are the predominant ras mutation found in pancreatic cancer [8,9], N-ras mutations predominate in acute myeloid leukemias [10], and H-ras mutations, which are generally rare, are most frequently observed in bladder cancer [11]. Oncogenic mutations of K-ras are involved in 20–50 % of sporadic colorectal carcinomas [12-14].

The association between human papillomavirus (HPV) infection and the development of cervical and anogenital tumors is widely accepted. However, the relationship between human papilloma viruses and malignant diesases at various body sites, including the upper respiratory and digestive tracts and the breast is still not clear [15-17]. HPV types 16 and 18 have been associated with a higher oncogenic potential and are considered as "high risk" types. The "low risk" HPV types HPV-6 and HPV-11 are predominantly associated with benign mucosal lesions of the genital tract and rarely result in invasive tumors [15,18]. The oncogenic HPV gene product E6 promotes degredation of the p53 tumor suppressor protein, whereas the E7 protein inactivates the Rb protein and related pocket proteins [19,20]. However, the tumorigenic properties of the E6 and E7 proteins may not necessarily be limited only to the Rb and p53-related pathways [21,22]. The presence of HPV infection alone also is not sufficient to cause tumorigenesis and requires additional cellular modifications such as alterations in the p53 and K-ras genes.

Although the role and distribution of K-ras mutations in colon cancer has been studied extensively there are no reports in the literature investigating the K-ras mutation status in HPV-associated colorectal cancer. The present study was undertaken to investigate the role of K-ras codon 12 and codon 13 mutations in HPV-associated colon tumors.

## Methods

Tumor samples were obtained at the time of surgery from 53 patients with colon cancer. The corresponding normal tissues surrounding the tumors were also analyzed. The study was approved by the Institutional Ethics Committee. Genomic DNA was extracted from the tumors and corresponding normal colon tissue samples by phenol/chloroform extraction. To detect K-ras codon 12 and 13 mutations, DNA was amplified by a mutagenic PCR assay. A mismatched upstream primer for codon 12 and a mismatched downstream primer for codon 13 which introduced a Bst N1 (codon 12) and a HaeIII (codon 13) restriction site in the wild type allele, respectively, were used for amplification as described previously [23]. The PCR reactions were carried out in a total volume of 25 μl containing 200 ng of genomic DNA, 50 mM KCl, 1.5 mM MgCl_2_, 10 pmol each of the forward and reverse primers, 1U Taq polymerase (MBI, Fermentas, Lithuania), 200 μM dNTP mix and 20 mM Tris-HCl, pH 8.3. The reaction mixture was heated to 95°C for 5 min. for initial denaturation, followed by 35 cycles of denaturation at 95°C for 1 min., annealing at 60°C for 1 min. and extension at 72°C for 1 min. Final extension was allowed to proceed for 6 min. at 72°C.

Digestion was performed in a total volume of 25 μl containing 10 μl PCR product, 2.5 μl 10x digestion buffer, 5 μg BSA and 10 U of Bst N1 or HaeIII by overnight incubation at 60°C or 37°C, respectively. The digested products were separated by 8% non-denaturing polyacrylamide gel electrophoresis at 120 V for 3 h, the gel was stained with ethidium bromide and the genotypes were determined using a video gel documentation system (Vilber-Lourmat, Marne-La-Vallée, France).

To investigate the HPV infection specific regions from the HPV genome were amplified by the MY 09 and MY 11 consensus primers which amplify a region of about 450 bp from the L1 open reading frame. DNA from a HPV-positive patient with cervix cancer was used as the positive control and DNA from lymphocytes were used as the negative control. The PCR reaction mix contained 500 mM KCl, 100 mM Tris-HCl, pH 9.0, 3 mM MgCl_2_, 200 μM of each dNTP, 150 ng sample DNA, 25 pmol of each primer and 2.5 U Taq polymerase. The reaction was begun by incubation at 95°C for five minutes followed by 35 cycles of denaturation at 95°C for 30 seconds, annealing at 55°C for 30 seconds and extension at 72°C for 1 minute.

30 μl of the PCR product was separated on a 1.5 % agarose gel and the DNA samples were blotted onto positive-charged nylon membranes (Boehringer Manheim, Germany). The probes used in the study were cloned into the pBR322 plasmid. They were separated from the vector by digestion with Bam H1 and Eco RI for HPV16 and HPV18, respectively. The probes were non-radioactively labeled at their 3'-end by incorporation of digoxigenin-labeled dideoxyuridine-triphosphate (DIG-ddUTP). Hybridization was performed at 68°C for 18 hours. The hybridized probes were visualized by an anti-DIG-alkaline phosphatase conjugate-catalyzed color reaction using BCIP (5-bromo-4-chloro-3-indolylphosphate) and nitroblue tetrazolium salt (Boehringer Manheim, Germany).

Statistical analyses were performed by using the chi-square and Fisher's exact tests with a significance level of p < 0.05.

## Results

To analyze the coexistence of human papillomavirus infection and K-ras gene activation in colon carcinoma, we investigated 53 tumor samples for the presence of HPV infection (types 6, 11, 16, 18 and 33) and K-ras codon 12 and codon 13 mutations. Codon 12 mutations were detected in 31 cases (58.5%) while fragments of the HPV genome were found in 43 (81.2%) cases. K-ras gene codon 13 mutations were not observed in the tumor samples of colon cancer patients. In 17 samples (32 %) genomic HPV fragments were also detected in the corresponding adjacent normal tissue. K-ras codon 12 or codon 13 mutations were not present in any of the normal tissue samples.

24 of 43 (55.8 %) HPV-positive samples and 7 of 10 (70%) HPV-negative samples analysed harbored a mutation in codon 12 of the K-ras gene. HPV 18 and HPV 33 were the most frequent types (73.6% and 56.6%, respectively). HPV 6, HPV 16, and HPV 11 were detected in 8, 5, and 2 cases, respectively. HPV 18 was not detected in the adjacent normal tissue samples. The most prevalent HPV types in normal tissue were HPV 16 (15%) and HPV 11 (13.2%). The distribution of HPV types between the normal and tumor tissue was not statistically significant except for HPV types 18 and 33 (p < 0.001).

Statistical analysis did not reveal an association between the HPV infection and K-ras mutation (Table 1). When we compared the frequencies of the K-ras mutations in high- and low-risk HPV-positive samples we failed to detect any correlation between the HPV type and K-ras mutation. We also found no correlation between the presence of K-ras codon 12 mutations and coinfection with more than one HPV type (Table 2).

## Discussion

The current genetic model for colon carcinogenesis depicts sequential accumulation of mutations in specific cancer-related genes, including APC, K-ras, and p53 that drive the transition from normal epithelium to increasing adenomatous dysplasia and finally to cancer [24,25]. During this process additional cofactors and modifications may act to immortalize and transform the cells. A great deal of evidence has shown that infection with specific papillomaviruses may be involved in the pathogenesis of malignant tumors at various body sites, including the anogenital area, the upper respiratory and digestive tracts, and the breast [15-17].

Numerous studies in the literature support the role of the ras protein and HPV viruses and indicate their probable cooperation in the pathogenesis of cervical neoplasias [26-28]. Mazurek et al [29] have shown that the E7 oncoprotein cooperates with ras in cell transformation. Directly binding to M2-PK and inducing its dimerization it thereby restores nucleic acid synthesis and cell proliferation.

The role of the ras oncogene [7,30-35] and HPV infection [36-38] in colorectal carcinomas has been investigated separately by several authors. However, the association between HPV infection and K-ras mutations has not been elucidated in colon cancer, yet.

In this study we observed mutations in the K-ras gene in 58.5 % of the tumor samples. This mutation frequency is slightly higher than the frequencies reported for the K-ras gene by other investigators in colon cancer [30-35,39]. K-ras mutations are thought to be an early event in colorectal neoplasia [1] and are observed in 9–10 % of small adenomas, in 40–50 % of large adenomas and in 40–65 % of colon carcinomas [24]. However, in a study analyzing K-ras mutations in four different series of colorectal cancer patients a mutation rate of 64 % was found in MYH-associated polyposis patients [39]. An even higher frequency of K-ras mutations (72 %) has been reported by Conlin et al [32], when colon cancer cases were grouped according to tumor stage. This variability can be explained by the selection of the patients and the methods used to analyse K-ras mutations. The studies mentioned above have sequenced the entire ras gene while we have investigated only the codon 12 and codon 13 mutations, which comprise 90% of the mutations in the ras gene [33] and 20–50% of the mutations observed in the colon cancer [12-14].

Bodaghi et al [37] reported that HPV infection is common in patients with colorectal cancer with the most prevalent type being HPV 16. However, the presence of HPV-DNA in colon tissue remains controversial. Although earlier studies [40,41] have failed to detect HPV-DNA in colon biopsy samples, more recent reports suggest that infection with HPV 16 and 18 may be etiologically associated with some cases of colon cancer [38,42]. In our study infection with HPV was observed in a significant proportion of the tumor samples. The high risk HPV 18 was the most prevalent HPV type. Interestingly, none of the adjacent normal tissue samples was positive for HPV 18. This observation is consistent with a recent report [38]. Our findings support the possibility that HPV infection is involved in colorectal carcinoma [36-38], as demonstrated by the fact that high risk HPV-DNA is present with a high frequency in malignant lesions [15,16].

## Conclusion

Our results suggest that mutational activation of the K-ras gene is common in colon carcinogenesis and infection with HPV may represent an additional important early step in the development of premalignant transformation. Further studies incorporating larger sample sizes are necessary to provide more definitive data on the potential role of these factors in colon carcinogenesis.

## Competing interests

The author(s) declare that they have no competing interests.

## Authors' contributions

NB conceived and participated in the design of the study, performed the experiments and drafted the manuscript. AT carried out the experiments. ND participated in the design and coordination of the study and revised the manuscript. All authors read and approved the final manuscript.

## Pre-publication history

The pre-publication history for this paper can be accessed here:



## Figures and Tables

**Table 1:** Distribution of the K-ras codon 12 mutations among samples infected with different types of HPV viruses.

Virus Type	K-ras mutation		No Mutation		Total	p
	n	%	n	%		
HPV 6	3	75	1	25	4	0.207
HPV 11	1	50	1	50	2	1
HPV 16	5	56	4	44	9	0.641
HPV 18	22	59	15	41	37	0.106
HPV 33	15	52	14	48	29	0.592
No Virus	7	70	3	30	10	0.09

**Table 2:** K-ras codon 12 mutation frequency according to the HPV risk types.

HPV type	K-ras mutation	No mutation	p
High risk	24	17	0.127
Low risk	16	14	0.611
Coinfection	7	3	0.09

## References

[B1] Fearon ER, Vogelstein BA (1990). A genetic model for colorectal tumorigenesis. Cell.

[B2] Barbacid M (1987). ras genes. Annu Rev Biochem.

[B3] Khosravi-far R, Der CJ (1994). The ras signal transduction pathway. Cancer Metastasis Rev.

[B4] Kiaris H, Spandidos DA (1995). Mutations of ras genes in human tumors. Int J Oncol.

[B5] Spandidos DA, Wilkie NM (1984). Malignant transformation of early passage rodent cells by a single mutated human oncogene. Nature.

[B6] Bos JL (1989). ras oncogen es in human cancer. A review. Cancer Res.

[B7] Russo A, Bazan V, Agnese V, Rodolico V, Gebbia N (2005). Prognostic and predictive factors in colorectal cancer: Kirsten Ras in CRC (RASCAL) and TP53CRC collaborative studies. Ann Oncol.

[B8] Soman NR, Wogan GN (1993). Activation of the c-Ki-ras oncogene in aflatoxin B1-induced hepatocellular carcinoma and adenoma in the rat: detection by denaturing gradient gel electrophoresis. Proc Nat Acad Sci.

[B9] Smit VT, Boot AJ, Smits AM, Fleuren GJ, Cornellisse CJ, Bos JL (1988). K-ras codon 12 mutations occur very frequently in pancreatic adenocarcinomas. Nucleic Acids Res.

[B10] Ahuja HG, Foti A, Bar-Eli M, Cline MJ (1990). The pattern of mutational involvement of RAS genes in human hematologic malignancies determined by DNA amplification and direct sequencing. Blood.

[B11] Buchill SA, Neal DE, Lunec J (1994). Frequency of H-ras mutations in human bladder cancer detected by direct sequencing. Br J Urol.

[B12] Bos JL, Fearon ER, Hamilton ER, Verlaan-de Vries M, van Boom JH, van der Eb AJ, Vogelstein B (1987). Prevalence of ras mutations in human colorectal cancers. Nature.

[B13] Boughdady IS, Kinsella AR, Haboubi NYK, Schofield PF (1992). K- ras gene mutation in adenomas and carcinomas of the colon. Surg Oncol.

[B14] Finkelstein SD, Sayeg R, Christensen S, Swalsky PA (1993). Genotipic classification of colorectal adenocarcinoma. Cancer.

[B15] zur Hausen H (1996). Papillomavirus infection-a major cause of human cancers. Biochim Biophys Acta.

[B16] zur Hausen H (2000). Papillomaviruses causing cancer : Evasion from host cell control in early events in carcinogenesis. J Natl Cancer Inst.

[B17] de Villiers EM, Sandstrom RE, zur Hausen H, Buck CE (2005). Presence of papillomavirus sequences in condylomatous lesions of the mamillae and in invasive carcinoma of the breast. Breast Cancer Res.

[B18] IARC (1998). Human papillomaviruses. IARC monographs on the evaluation of carcinogenic risks to humans.

[B19] Dyson N, Howley PM, Munger K, Harlow E (1989). The human papillomavirus-16 E7 oncoprotein is able to bind to the retinoblastoma gene product. Science.

[B20] Scheffner M, Münger K, Byrne JC, Howley PM (1991). The state of the p53 and retinoblastoma genes in human cervical carcinoma cell lines. Proc Natl Acad Sci USA.

[B21] Münger K, Basile JR, Duensing S, Eichten A, Gonzales SL, Grace M, Zacny VL (2001). Biological activities and molecular targets of the human papillomavirus E7 oncoprotein. Oncogene.

[B22] Mantovani F, Banks L (2001). The human papillomavirus E6 protein and its contribution to malignant progression. Oncogene.

[B23] Hatzaki A, Razi E, Anagnostopoulou K, Iliadis K, Kodaxis A, Papaioannou D, Labropoulos S, Vasilaki M, Kosmidis P, Saetta A, Mihalatos M, Nasioulas G (2001). A modified mutagenic PCR-RFLP method for K-ras codon 12 and 13 mutations detection in NSCLC patients. Mol Cell Probes.

[B24] Vogelstein B, Fearon ER, Hamilton SR, Kern SE, Preisinger AC, Leppert M, Nakamura Y, White R, Smits AM, Bos JL (1988). Genetic alterations during colorectal-tumor development. N Engl J Med.

[B25] Piard F, Chapusot C, Ecarnot-Laubriet A, Ponnelle T, Martin L (2002). Molecular markers of heterogeneity in colorectal cancer and adenomas. Eur J Cancer Prev.

[B26] Mouron SA, Abba MC, Güerci A, Gomez MA, Dulout FN, Golijow CD (2000). Association between activated K-ras and c-erbB-2 oncogenes with "high risk" and "low risk" human papillomavirus types in preinvasive cervical lesions. Mutat Res.

[B27] Prokopakis P, Sourvinos G, Koumantaki Y, Koumantakis E, Spandidos DA (2002). K-ras mutations and HPV infection in cervicitis and intraepithelial neoplasias of the cervix. Oncol Rep.

[B28] Pochylski T, Kwasniewska A (2003). Absence of point mutation in codons 12 and 13 of K-ras oncogene in HPV-associated high grade dysplasia and squamous cell cervical carcinoma. Eur J Obstet Gynecol Reprod Biol.

[B29] Mazurek S, Zwerschke W, Jansen-Durr P, Eigenbrodt E (2001). Metabolic cooperation between different oncogenes during cell transformation: interaction between activated ras and HPV-16 E7. Oncogene.

[B30] Kornaros S, Liloglou T, Politou M, Elemenoglou I (2005). Colorectal adenomas with a low frequency of ki-ras mutations: a sample from a population with a low mortality rate from colorectal cancer. Colorectal Dis.

[B31] Brink M, Weijenberg MP, deGoeij AFPM, Roemen GMJM, Lentjes, Bruiine AP, Goldbohm RA, van der Brandt PA (2005). Meat consumption and K-ras mutations in sporadic colon and rectal cancer in The Netherlands cohort study. Br J Cancer.

[B32] Conlin A, Smith G, Corey FA, Wolf CR, Steele RJC (2005). The prognostic significance of K-ras, p53 and APC mutations in colorectal carcinoma. Gut.

[B33] Bazan V, Agnese V, Corsale S, Calo V, Valerio MR, Latteri MA, Vieni S, Grassi N, Cicero G, Dardanoni G, Tomasino RM, Colucci G, Gebbia NA, Russo A (2005). Specific TP53 and/or Ki-ras mutations as independent predictors of clinical outcome in sporadic colorectal adenocarcinomas: results of a 5-year Gruppo Oncologico dell'Italia Meridionale (GOIM) prospective study. Ann Oncol.

[B34] Hsieh JS, Lin SR, Chang MY, Chen FM, Lu CY, Huang TJ, Huang YS, Huang CJ, Wang JY (2005). APC, K-ras, and p53 gene mutations in colorectal cancer patients: Correlation to clinicopathologic features and postoperative surveillance. Am Surg.

[B35] Wu CM, Tang R, Wang JY, Changchien CR, Hsieh LL (2005). Frequency and spectrum of K-ras codons 12 and 13 mutations in colorectal adenocarcinomas from Taiwan. Cancer Genet Cytogenet.

[B36] Buyru N, Budak M, Yazici H, Dalay N (2003). p53 gene mutations are rare in human papillomavirus-associated colon cancer. Oncol Rep.

[B37] Bodaghi S, Yamanegi K, Xiao SY, Da Costa M, Palefsky JM, Zheng ZM (2005). Colorectal papillomavirus infection in patients with colorectal cancer. Clin Cancer Res.

[B38] Perez LO, Abba MC, Laguens RM, Golijow CD (2005). Analysis of adenocarcinoma of the colon and rectum: detection of human papillomavirus (HPV) DNA by polymerase chain reaction. Coleractal Dis.

[B39] Johnson V, Lipton LR, Cummings C, Eftekhar Sadat AT, Izatt L, Hodgson SJ, Talbot IC, Thomas HJ, Silver AJ, Tomlinson IP (2005). Analysis of somatic molecular changes, clinicopathological features, family history and germline mutations in colorectal cancer families: evidence for efficient diagnosis of HNPCC and for distinct groups of non-HNPCC families. J Med Genet.

[B40] Shroyer KR, Kim JG, Manos MM, Greer CE, Pearlman NW, Franklin WA (1992). Papillomavirus found in anorectal squamous carcinoma, not in colon adenocarcinoma. Arch Surg.

[B41] Shah KV, Daniel RW, Simons JW, Vogelstein B (1992). Investigation of colon cancers for human papillomavirus genomic sequences by polymerase chain reaction. J Surg Oncol.

[B42] Weinberger PM, Yu Z, Zerkowski M, Chung G, Camp RL, Rimm DL, Psyrri A (2004). A possible association of human papillomavirus with a subset of colorectal adenocarcinomas. J Clin Oncol.

